# Field-effect control of superconductivity and Rashba spin-orbit coupling in top-gated LaAlO_3_/SrTiO_3_ devices

**DOI:** 10.1038/srep12751

**Published:** 2015-08-05

**Authors:** S. Hurand, A. Jouan, C. Feuillet-Palma, G. Singh, J. Biscaras, E. Lesne, N. Reyren, A. Barthélémy, M. Bibes, J. E. Villegas, C. Ulysse, X. Lafosse, M. Pannetier-Lecoeur, S. Caprara, M. Grilli, J. Lesueur, N. Bergeal

**Affiliations:** 1 Laboratoire de Physique et d’Etude des Matériaux -CNRS-ESPCI ParisTech-UPMC, PSL Research University, 10 Rue Vauquelin, 75005 Paris, France; 2 Unité Mixte de Physique CNRS-Thales, 1 Av. A. Fresnel, 91767 Palaiseau, France; 3 Laboratoire de Photonique et de Nanostructures LPN-CNRS, Route de Nozay, 91460 Marcoussis, France; 4 DSM/IRAMIS/SPEC - CNRS UMR 3680, CEA Saclay, F-91191 Gif sur Yvette Cedex, France; 5 Dipartimento di Fisica Università di Roma “La Sapienza”, piazzale Aldo Moro 5, I-00185 Roma, Italy

## Abstract

The recent development in the fabrication of artificial oxide heterostructures opens new avenues in the field of quantum materials by enabling the manipulation of the charge, spin and orbital degrees of freedom. In this context, the discovery of two-dimensional electron gases (2-DEGs) at LaAlO_3_/SrTiO_3_ interfaces, which exhibit both superconductivity and strong Rashba spin-orbit coupling (SOC), represents a major breakthrough. Here, we report on the realisation of a field-effect LaAlO_3_/SrTiO_3_ device, whose physical properties, including superconductivity and SOC, can be tuned over a wide range by a top-gate voltage. We derive a phase diagram, which emphasises a field-effect-induced superconductor-to-insulator quantum phase transition. Magneto-transport measurements show that the Rashba coupling constant increases linearly with the interfacial electric field. Our results pave the way for the realisation of mesoscopic devices, where these two properties can be manipulated on a local scale by means of top-gates.

The interplay between superconductivity and spin-orbit coupling (SOC) is at the centre of intensive research efforts as it can generate a variety of unique phenomena such as the occurrence of triplet superconductivity, for instance^1^. Recently, hybrid nanostructures involving a superconductor in proximity to a semiconducting nanowire with a strong SOC have been proposed as an ideal system to observe a topological superconducting phase, which accommodates pairs of Majorana fermions^2^,^3^. Following this idea, the first signatures of Majorana Fermions were obtained in devices made with indium antimonide in contact with niobium titanium nitride^4^. However, the realisation of such devices remains a challenge because (i) the intrinsic value of the SOC in semiconductors is weak and cannot be tuned (ii) it is difficult to control the spin state at the interface between very different materials. For this reason, the discovery of a two-dimensional electron gas (2-DEG) at the interface between two insulating oxides such as LaAlO_3_/SrTiO_3_ or LaTiO_3_/SrTiO_3_ raised a considerable interest^5^. Indeed, this 2-DEG displays both superconductivity^6^,^7^ and a strong SOC which is expected to be Rashba-type^8^,^9^, a combination of properties which is rarely observed in the same material.

The 2-DEG whose typical extension in the SrTiO_3_ substrate is of order ~10 nm^10^,^12^ is confined in an interfacial quantum well buried under an few unit cells thick insulating LaAlO_3_ layer. By adjusting the Fermi level with a gate voltage, the conductivity of the 2-DEG can be modulated from insulating to superconducting^11^,^12^. In addition, the Rashba SOC, which is dominated by the local electric field at the interface, can also be controlled with a gate voltage^8^. The combination of these two effects enables the realisation of nanostructures, where the very same material can be turned into different states by applying a local electric field-effect. Thus far, controlling the superconductivity and SOC have been demonstrated almost exclusively with gates deposited at the back of thick SrTiO_3_ substrates. Because of the very high value of the SrTiO_3_ dielectric constant at low temperatures (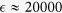)^13^, the electric field-effect can significantly modulate the carrier density with gate voltages on the order of 100 V^11^,^12^,^14^. However, in such geometry, it is not possible to control the properties of the 2-DEG on a scale much smaller than the typical thickness of the substrate (500 *μ*m), making it impossible to realise devices with dimensions comparable to lengths that are characteristic of quantum orders (such as the superconducting coherence length and the spin diffusion length). To overcome this problem, field-effect control of the superconductivity and Rashba SOC needs to be achieved by means of local top-gates. Forg *et al.* fabricated field-effect transistors in a LaAlO_3_/SrTiO_3_ heterostructures using the insulating LaAlO_3_ layer as the gate dielectric and the YBa_2_Cu_3_O_7_ layer as the top-gate electrode^15^. Hosoda *et al.* achieved top-gate control of the normal state properties using a metallic gate directly deposited on the LaAlO_3_ layer^16^. More recently, a first attempt to modulate the superconductivity with a top-gate gave promising results^17^, despite the leaky insulating LaAlO_3_ layer. In this article, the realisation of a top-gated field-effect device is reported. The properties of the 2-DEG could be tuned over a wide range, from a superconducting to an insulating state. In addition, the control of the Rashba SOC by means of a top-gate is also demonstrated.

A ten-*μm*-wide superconducting Hall bar was first fabricated with an amorphous LaAlO_3_ template method and then covered by a Si_3_N_4_ dielectric layer and a metallic top gate (see Fig. 1a,b)^18^. More information on the fabrication processes is given in the Methods section. The sample was anchored to the mixing chamber of a dilution refrigerator with a base temperature of 16 mK. Figure 1c shows the superconducting transition of the device at the critical temperature 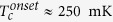, which is similar to an unprocessed LaAlO_3_/SrTiO_3_ heterostructure. The current-voltage (I-V) characteristics of the device abruptly switches from the superconducting state (*R* = 0) to the resistive state (*R *≠ 0) at the critical current *I*_*c*_ = 460 nA which corresponds to a critical current density of approximately 500 *μ*A/cm.

## Electrostatic Control of the Carrier Density

**Figure 2:**
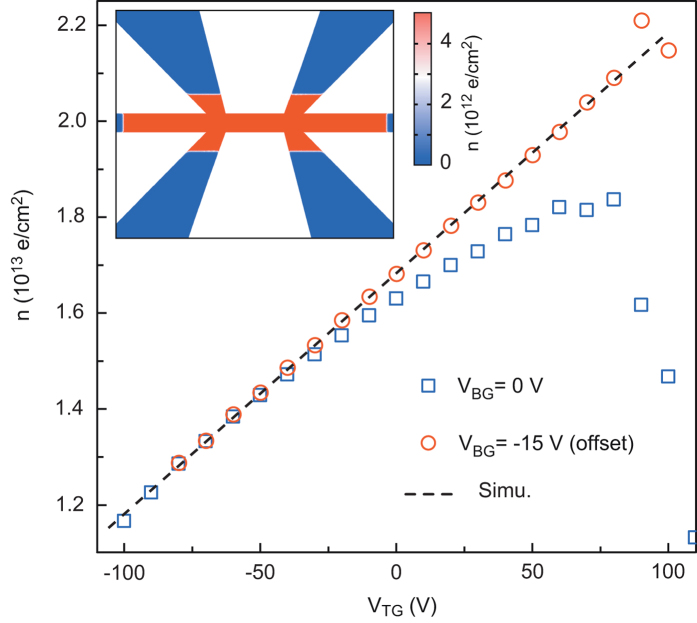
Hall effect and carrier density. Carrier density (*n*) extracted from the slope of the Hall voltage (*V*_*H*_) at 4 T as a function of *V*_TG_ for two different back-gate voltages (*V*_BG_). The curve at *V*_BG_ = −15 V is offset to match the curve at *V*_BG_ = 0 V at negative top-gate voltages. The dashed line was obtained from numerical simulations on the carrier density, assuming a dielectric constant 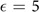 for the Si_3_N_4_ layer. Inset: example of a numerical simulation of the charge carrier distribution in the device for *V*_BG_ = 0 V and *V*_TG_ = 10 V.

After the sample was cooled, the top-gate voltage *V*_TG_ was first increased to +110 V, beyond the saturation threshold of the resistance. During this operation, electrons are added in the quantum well, increasing the Fermi energy to its maximum value (i.e., the top of the well)^19^. In comparison with back-gate experiments where the relationship between the carrier density (*n*) and the back-gate voltage *V*_BG_ is not trivial owing to the electric-field-dependent dielectric constant of SrTiO_3_^13^, here, the carrier density is expected to increase linearly with *V*_TG_. Figure 2 shows the sheet carrier density 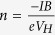, extracted from the Hall effect measurements performed up to B = 4 T as a function of the top-gate voltage *V*_TG_, for two different back-gate voltages (*V*_BG_ = 0 V and *V*_BG_ = −15 V). For *V*_BG_ = 0 V, the linear increase in *n* is observed with *V*_TG_ only for negative *V*_TG_. The non-physical decrease in *n* with *V*_TG_ for positive gate voltages is caused by the incorrect determination of the carrier density at low magnetic fields. It was shown that at the LaAlO_3_/SrTiO_3_ interface, the Hall voltage is no longer linear with the magnetic field for strong filling of the quantum well because of multi-band transport^12^,^20^,^21^. To reach a doping regime where the one-band approximation is valid, a negative back-gate *V*_BG_ = −15 V was applied producing a depletion of the highest energy sub-bands that accommodate the highly-mobile carriers, responsible of the decrease of the Hall number at positive *V*_TG_. Figure 2 shows that in this case, the linear dependence of 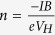 with *V*_TG_ can be recovered. The linear fit of slope 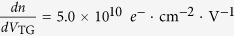 is obtained from numerical simulations of the electric field-effect by a finite elements method assuming a dielectric constant 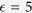 for the Si_3_N_4_ layer (see the inset in Fig. 2). Finally, the following relationship between the carrier density and top-gate voltage is deduced: *n* = 5.0 × 10^10^ *V*_TG_ + 1.69 × 10^13^ e^-^ .cm^-2^.

## Superconductivity and Phase Diagram

**Figure 3:**
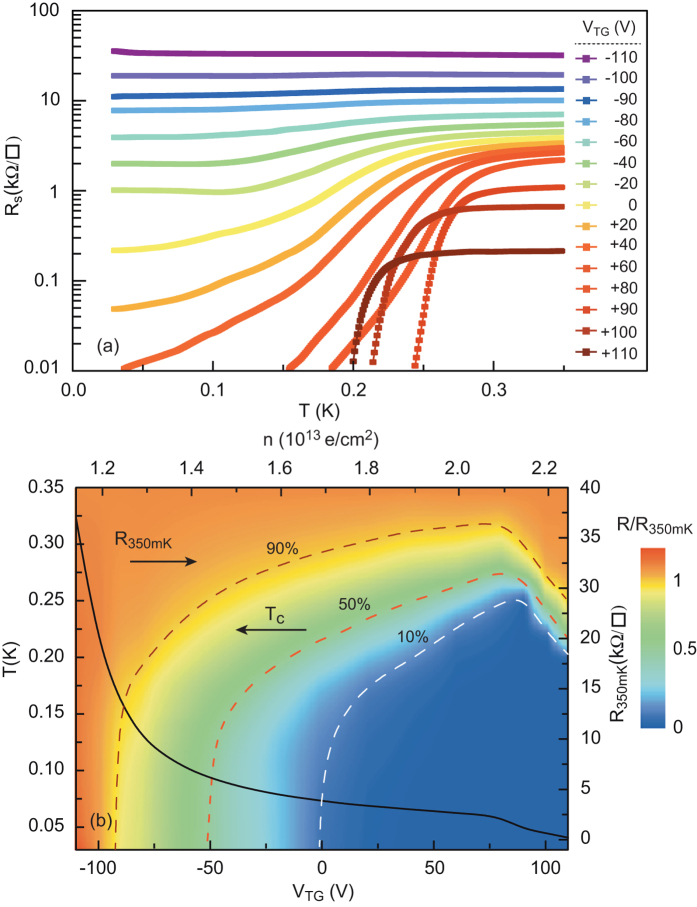
Field-effect control of the superconductivity. (**a**) Sheet resistance of the device as a function of temperature for different *V*_TG_. (**b**) Sheet resistance normalised by its value at T = 350 mK plotted with a colour scale as a function of temperature (left axis) and top-gate voltage. The carrier densities corresponding to the top-gate voltages have been added in the top axis. The sheet resistance at T = 350 mK is plotted as a function of top-gate voltage on the right axis. The critical temperature T_*c*_ is plotted as function of the top-gate voltage on the left axis for the different criteria: drop of 10%, 50% and 90% of the normal resistance taken at T = 350 mK.

In the following, the back gate voltage *V*_BG_ was always set to 0 V unless otherwise stated. Figure 3a shows the sheet resistance of the device as a function of temperature measured for different top-gate voltages in the range [−110 V, +110 V], where the leakage gate current is negligible (<0.1 nA). The variation in *V*_TG_ induces a modulation in the normal state resistance by two orders of magnitude. Figure 3b summarises the variations of the normalised resistance *R*/*R*(*T* = 350 mK) as a function of temperature (*T*) and top-gate voltage *V*_TG_ on a phase diagram. The corresponding *n* is also indicated on the top axis. The device displays a gate-dependent superconducting transition, whose critical temperature *T*_*c*_ describes a partial dome as a function of *V*_TG_, similar to that observed with a back-gate^11^,^12^,^14^. The maximum *T*_*c*_, corresponding to optimal doping, is around 250 mK. In the underdoped region, a decrease in the gate voltage causes *T*_*c*_ to continuously decrease from its maximum value to zero. A superconductor-to-insulator quantum phase transition takes place around *V*_TG_ = −90 V. The critical sheet resistance at the transition is 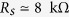, which is close to the quantum of resistance of bosons with 2e charges, 
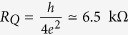
. For large negative voltages, corresponding to low electron densities, the sheet resistance increases strongly when approaching the insulating state. In the overdoped region, the addition of electrons into the quantum well with the top-gate produces a small decrease in *T*_*c*_ whose origin is currently under debate. Such behaviour has also been observed in doped bulk SrTiO_3_^22^ and could be reinforced by the two-dimensionality of the interface^23^. The current-voltage characteristics of the device for different top-gate voltages are shown in Supplementary Material.

## Rashba Spin-orbit Coupling

In LaAlO_3_/SrTiO_3_ heterostructures, the accumulation of electrons in the interfacial quantum well generates a strong local electric field *E*_*z*_ perpendicular to the motion of the electrons, which translates into a magnetic field in their rest frame. It is expected that the coupling of the electrons spin to this field gives rise to a Rashba-type SOC described by the Hamiltonian 
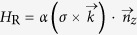
, where 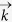 is the electron wave vector, 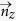 is a unit vector perpendicular to the interface and *σ* are the Pauli matrices^24^. The constant *α* represents the strength of the SOC and has to be directly proportional to the interfacial electric field *E*_*z*_. In electronic transport measurements, the presence of a spin-orbit coupling results in an additional spin relaxation mechanism characterised by the relaxation time *τ*_SO_. Caviglia *et al.* reported a *τ*_SO_ roughly proportional to the inverse of the elastic scattering time *τ*_*e*_ in agreement with a D’Yakonov-Perel mechanism characteristic of a Rashba interaction^8^. However, to confirm experimentally the Rashba-type SOC, it is also important to establish the linear dependance of *α* with *E*_*z*_ (*α* ∝ *E*_*z*_) when the filling of the quantum well is varied by gating.

**Figure 4:**
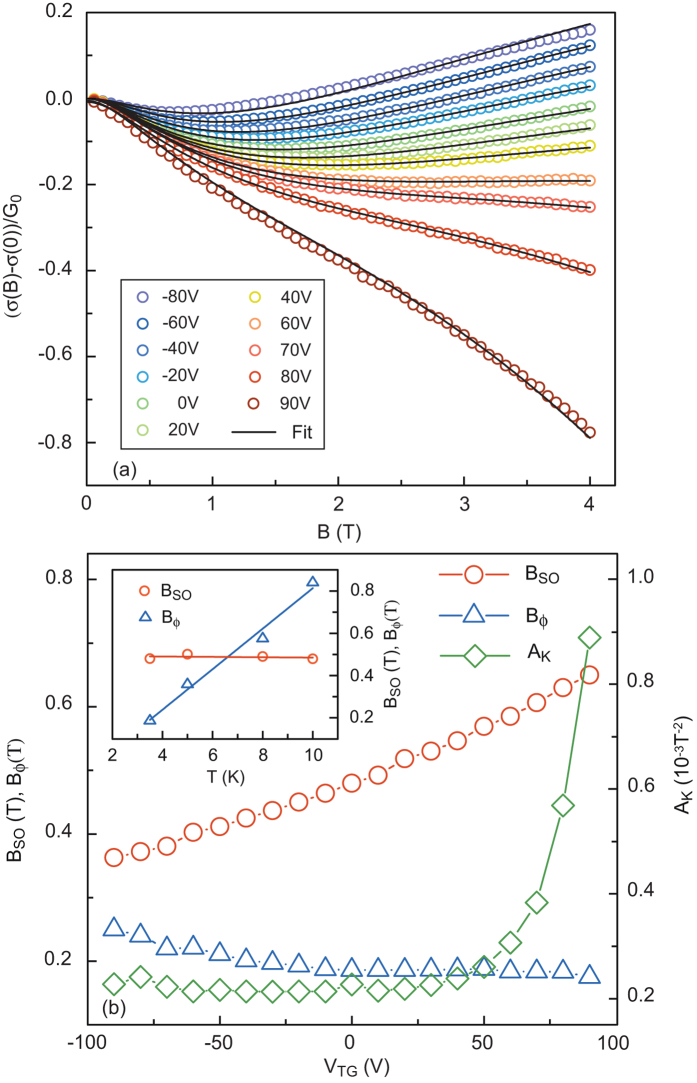
Magnetotransport measurements. (**a**) Magnetoconductance of the device at T = 3.5 K for different *V*_TG_. The experimental data (open symbols) are fitted with the Maekawa-Fukuyama formula (1). (**b**) Evolution of the fitting parameters *B*_SO_, *B*_*ϕ*_ and *A*_*K*_ as a function of the gate voltage. Inset) Variations in *B*_SO_ and *B*_*ϕ*_ as a function of temperature for *V*_TG_ = 0.

The weak localization corrections to the conductance of a two-dimensionnal system at low temperatures are modified by the presence of an additional spin relaxation mechanism due to SOC^25^,^26^ whose strength can therefore be determined by properly analysing the magnetoconductance Δ*σ*(*B*) = *σ*(*B*) − *σ*(0). Δ*σ*(*B*) was measured in the normal state at different temperatures and top-gate voltages. For negative *V*_TG_ a positive magnetoconductance was observed beyond 1 T. This is characteristic of a weak localization regime with small SOC (Fig. 4). As *V*_TG_ is increased, an inversion of the sign of the magnetoconductance is observed and at large positive gate voltages the magnetoconductance remains always negative. The experimental data in Fig. 4 were fitted with the Maekawa-Fukuyama formula in a diffusive regime that describes the change in the conductivity with magnetic field with negligible Zeeman splitting^25^,


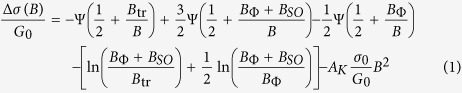


where Ψ is the digamma function, 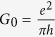 is the quantum of conductance, and the parameters *B*_tr_, *B*_Φ_, *B*_SO_ are the effective fields related to the elastic, inelastic and spin-orbit relaxation times respectively. *B*_Φ_ and *B*_SO_, which are measured here by a transport experiment, are related to the relaxation times *τ*_Φ_ and *τ*_SO_ by the expressions 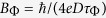 and 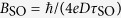 respectively, where *D* is the diffusion constant^25^,^26^. Finally, to account for the orbital magnetoconductance, we have added in Eq. (1) a *B*^2^ term with a Kohler coefficient *A*_*K*_ which increases quadratically with the mobility^27^,^28^. Good agreement is obtained between the experimental data and the theory over the whole electrostatic doping range.

The evolution of the fitting parameters as a function of the top-gate voltage and equivalent carrier density is shown in Fig. 4b. *B*_*ϕ*_ varies only weakly over the whole range of gate voltage, indicating that the number of inelastic collisions does not depend on the carrier density. In the framework of the weak localisation theory the temperature dependence of the inelastic scattering time is given by *τ*_Φ_ ∝ *T*^−*p*^ and therefore *B*_Φ_ ∝ *T*^*p*^, where *p* depends on the inelastic mechanism. The same fitting procedure was performed at different temperatures, giving a linear relationship between *B*_Φ_ and *T* (Fig. 4b inset and Supplementary Material). This is consistent with *p* = 1, which indicates that the inelastic scattering is dominated by electron-electron interactions^6^,^29^.

## Spin-splitting Energy

**Figure 5:**
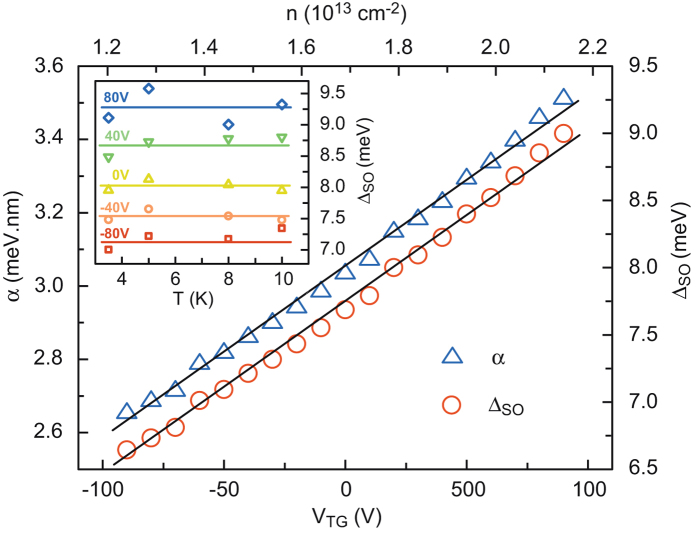
Spin-orbit splitting. Spin-orbit splitting as a function of *V*_TG_ (bottom axis) and corresponding carrier density (top axis). Inset: Spin-orbit splitting as a function of temperature for selected *V*_TG_.

The spin-orbit term (*B*_SO_) increases with top-gate voltage and, correspondingly, with the carrier density. The analysis of this dependence can shed light on the origin of the SOC at the LaAlO_3_/SrTiO_3_ interface. If we assume that the spin relaxation is dominated by the D’Yakonov-Perel mechanism, based on a Rashba spin-orbit interaction, 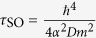
26,30. We then obtain the relationship between the coupling constant and the spin-orbit effective field 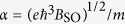. Integrating the Maxwell-Gauss equation in the direction perpendicular to the interface gives the interfacial electric field 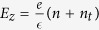 where 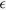 is the dielectric constant of Si_3_N_4_ at the interface and *n*_*t*_ is the carrier density of non-mobile charges trapped in the SrTiO_3_ substrate. The coupling constant being proportional to *E*_*z*_, it is therefore expected to vary with carrier density with the form *α* = *an* + *b*, which is well satisfied experimentally for a wide range of electrostatic doping (Fig. 5). This confirms experimentally that the D’Yakonov-Perel mechanism in the presence of Rashba spin-orbit interaction is dominant in these 2-DEGs.

Assuming a Fermi energy of 100 meV and an effective mass *m* = 0.7*m*_0_ for the *d*_*xy*_ light subbands mainly occupied, we can estimate the characteristic spin-splitting energy Δ_SO_ = 2*k*_*F*_*α* where *k*_*F*_ is the electron wave vector at Fermi energy. The order of magnitude of a few meV, which is much larger than in most semiconductors, is in agreement with previous studies^8^,^9^. Neglecting the small changes in *k*_*F*_ with doping, we can plot the variation of Δ_SO_ with *V*_TG_, and correspondingly, *n* (Fig. 5). Δ_SO_ is independent of the temperature below 10 K as the shape of the quantum well and *E*_*z*_ do not change in this temperature range (see the inset in Fig. 5 and Supplementary Material). The Kohler term (parameter *A*_*K*_) is proportional to the square of the mobility. For positive gate voltages where the 2-DEG has a rather large mobility, this term dominates the magnetoconductance and must be taken into account in Eq. (1). As shown in the Supplementary Figure S4, fitting the data without this term leads to an incorrect determination of the SOC in a large range of positive gating.

In summary, LaAlO_3_/SrTiO_3_ -based field-effect devices were fabricated using the amorphous LaAlO_3_ template method. The superconductivity can be electrostatically modulated over a wide range by a top-gate voltage, without any leakage. A superconductor-to-insulator quantum phase transition is induced when the quantum well is strongly depleted. By analysing the magnetotransport measurements, the presence of strong spin-orbit coupling that could be controlled with the top-gate voltage was demonstrated. The spin-spliting energy on the order of a few meV was found to increase linearly with the interfacial electric field in agreement with the Rashba mechanism. These results represent an important step toward the realisation of new mesoscopic devices, where the interaction between superconductivity and the Rasba SOC could give rise to non-conventional electronic states.

## Methods

### Device fabrication

Starting with a TiO_2_ -terminated -oriented SrTiO_3_ commercial substrate (Crystec), the template of a Hall bar with contact pads was defined by evaporating an amorphous LaAlO_3_ layer through a resist patterned by optical lithography. After a lift-off process, a thin layer of crystalline LaAlO_3_ (8 u.c) was grown on the amorphous template by Pulse Laser Deposition, such that only the areas directly in contact with the substrate (Hall bar and contact pads) were crystalline. A KrF excimer (248 nm) laser was used to ablate the single-crystalline LaAlO_3_ target at 1 Hz, with a fluence between 0.6 and 1.2 J/cm^2^ under an O_2_ pressure of 2 × 10^−4^ mbar^31^. The substrate was typically kept at 650 °C during the growth of the film, monitored in real-time by reflection high-energy electron diffraction RHEED. As the growth occurs layer-by-layer, the thickness can be controlled at the unit cell level. After the growth of the film, the sample was cooled down to 500 °C under a O_2_ pressure of 10^−1^ mbar, which was increased up to 400 mbar. To reduce the presence of oxygen vacancies (in both the substrate and the film), the sample was kept under these conditions for 30 minutes before it was cooled to room temperature. The 2-DEG forms at the interface between the crystalline LaAlO_3_ layer and the SrTiO_3_ substrate. Such method has already been used to fabricate ungated 500 nm wide channels without noticeable alteration of the 2DEG properties^18^. Once the channel is defined, a 500 nm thick Si_3_N_4_ dielectric layer was deposited on the Hall bar by a lift-off process. After this step, a gold top-gate layer was deposited and lifted-off forming and appropriate geometry to cover the Hall bar. A metallic back gate was added at the end of the process.

## Additional Information

**How to cite this article**: Hurand, S. *et al.* Field-effect control of superconductivity and Rashba spin-orbit coupling in top-gated LaAlO_3_\SrTiO_3_ devices. *Sci. Rep.* **5**, 12751; doi: 10.1038/srep12751 (2015).

## Supplementary Material

Supplementary Information

## Figures and Tables

**Figure 1:**
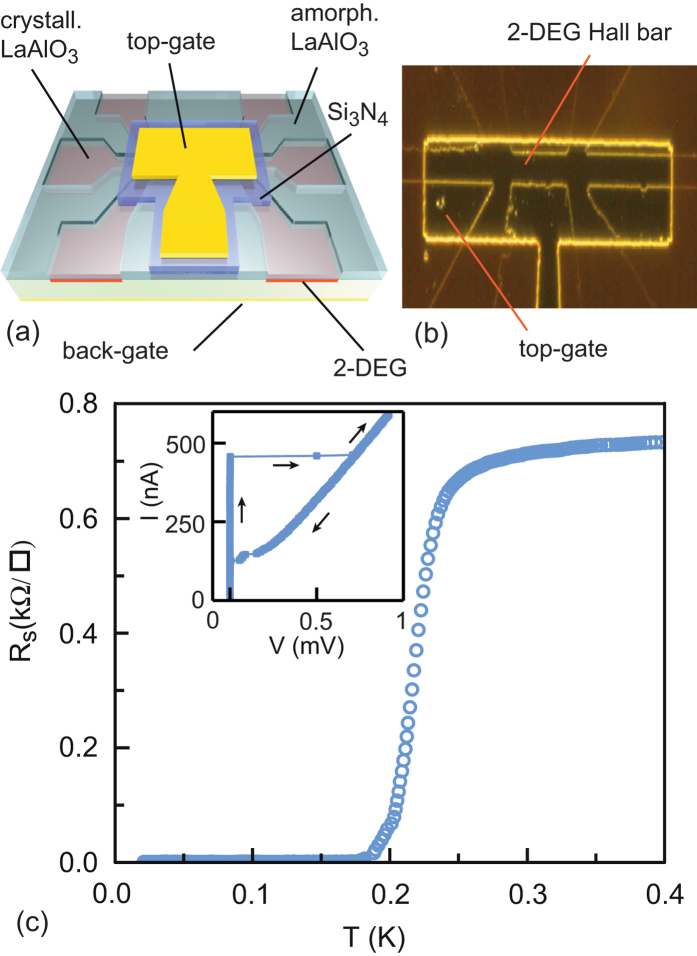
Device structure and superconducting transition. (**a**) Schematic of the LaAlO_3_/SrTiO_3_ device with a 500 nm thick Si_3_N_4_ dielectric layer (scheme drawn by N. B.). (**b**) Dark-field optical picture of the device showing the Hall bar covered by a top-gate. (**c**) Sheet resistance as a function of temperature showing a superconducting transition at a critical transition temperature *T*_*c*_ ≈ 250 mK. Inset) Current-voltage characteristics of the device indicating the critical current *I*_*c*_ = 460 nA. The arrows indicate the direction of the current sweep.
