# Supplementary material for: Field-effect control of superconductivity and Rashba spin-orbit coupling in top-gated LaAlO3/SrTiO3 devices
Source: Sci Rep. 2015 Aug 5;5:12751. doi: 10.1038/srep12751 (PMC4525493; doi:10.1038/srep12751)
Supplement: Supplementary Information [file srep12751-s1.pdf]

Supplementary Material

Field-effect control of superconductivity and Rashba spin-orbit coupling in top-gated $LaAlO_3/SrTiO_3$ devices

S. Hurand[1], A. Jouan[1], C. Feuillet-Palma[1], G. Singh[1], J. Biscaras[1], E. Lesne[2], N. Reyen[2], A. Barthélémy[2], M. Bibes[2], J. E. Villegas[2], C. Ulysse[3], X. Lafosse[3], M. Pannetier-Lecoeur[4], S. Caprara[5], M. Grilli[5], J. Lesueur[1], N. Bergeal[1]

[1]*Laboratoire de Physique et d'Etude des Matériaux -CNRS-ESPCI ParisTech-UPMC, PSL Research University, 10 Rue Vauquelin - 75005 Paris, France.*

[2]*Unité Mixte de Physique CNRS-Thales, 1 Av. A. Fresnel, 91767 Palaiseau, France*

[3]*Laboratoire de Photonique et de Nanostructures LPN-CNRS, Route de Nozay, 91460 Marcoussis, France*

[4]*DSM/IRAMIS/SPEC - CNRS URA 3680, CEA Saclay, F-91191 Gif sur Yvette Cedex, France and*

[5]*Dipartimento di Fisica Università di Roma"La Sapienza", piazzale Aldo Moro 5, I-00185 Roma, Italy*

(Dated: April 30, 2015)

I. Current-voltage characteristics of the device

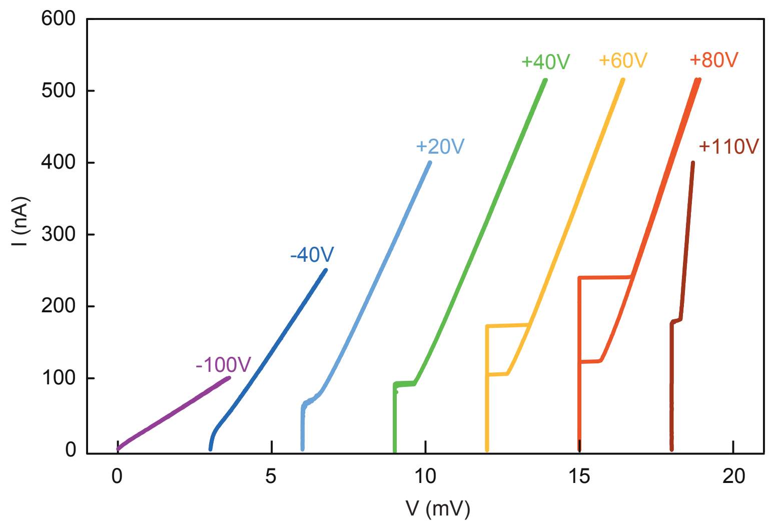

Figure S1: Current-voltage characteristics of the device for different values of V_{TG}.

Supplementary Figure 1 shows the current-voltage (I-V) characteristics of the device for different values of the top-gate voltage V_{TG}. Curves display an abrupt switching from the superconducting state ($R = 0$) to the resistive state ($R \neq 0$) at the critical current as already already observed in $LaAlO_3/SrTiO_3$ heterostructures [1, 2]. When the current is decreased from $I > I_c$ the system gets retrapped in the superconducting state at a lower current. The presence of both the abrupt switching and the hysteresis tends to indicate that the superconductor behave as a Josephson junction array rather than an homogeneous superconductor.

II. Magneto-transport measurements at different temperatures

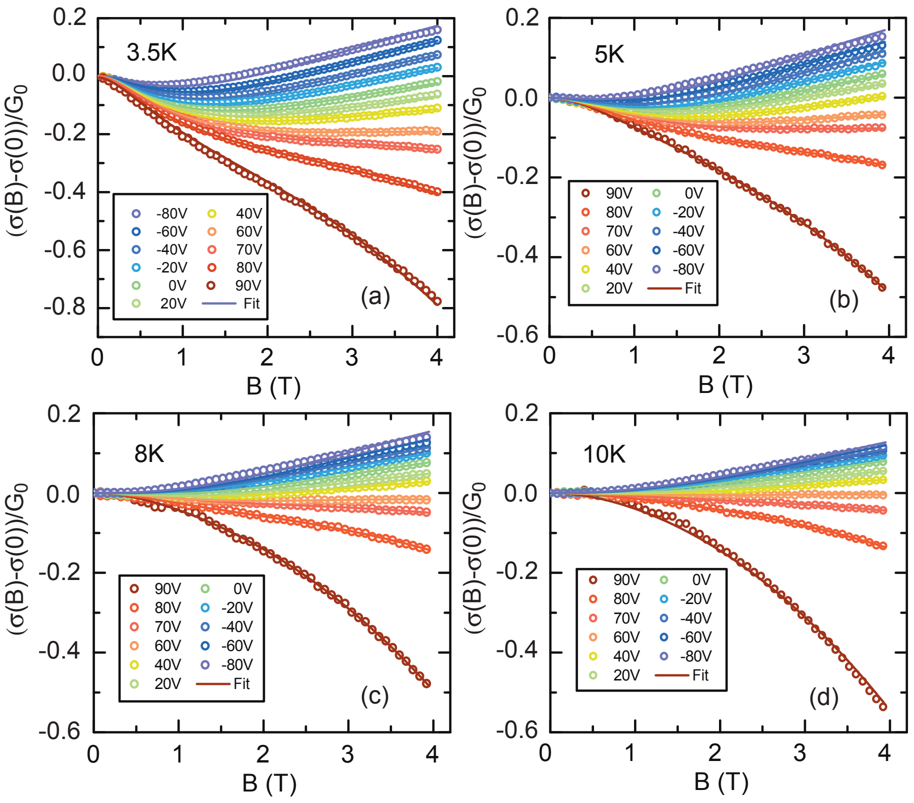

Figure S2: a) Magneto-conductance of the device for different values of V_{TG} measured for four different temperatures, T=3,5K (panel a), T=5K (panel b), T=8K (panel c), T=10K (panel d). Experimental data (open symbols) are fitted by the Maekawa-Fukuyama formula (**??**) described in the article.

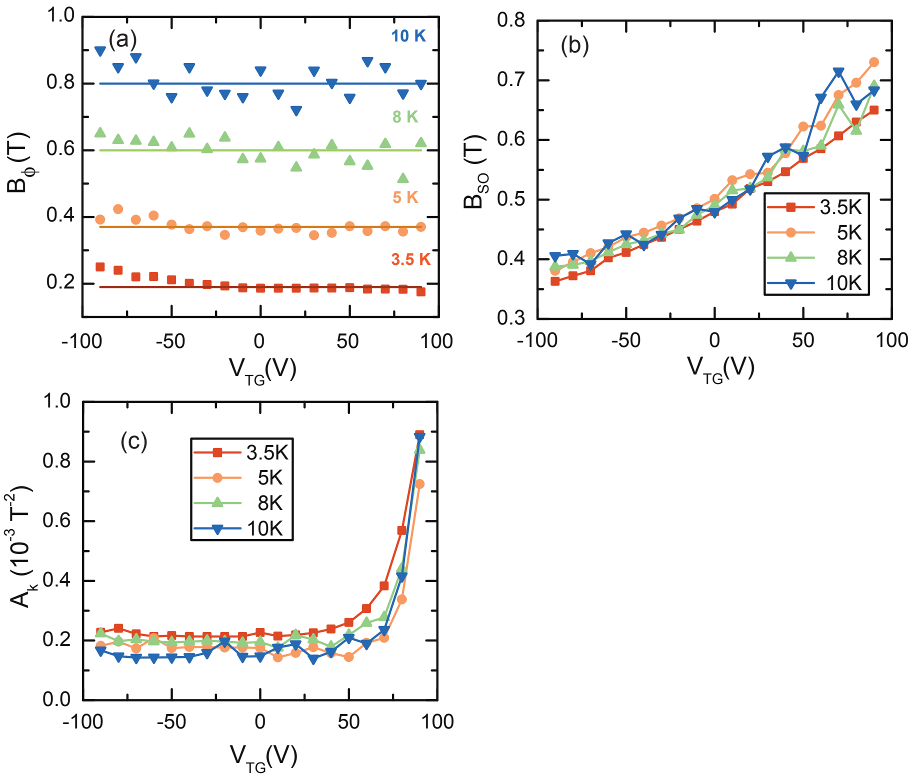

Figure S3: Evolution of the fitting parameters B_ϕ (panel a), B_{SO} (panel b) and A_K (panel c) as a function of V_{TG} for different temperatures.

We measured the magneto-conductance $\Delta\sigma(B)$ in the normal state for different temperatures and top-gate voltages. The experimental data of Supplementary Figure 2 were fitted with the Maekawa-Fukuyama formula for a diffusive regime that describes the change of conductivity with magnetic field $\Delta\sigma(B) = \sigma(B) - \sigma(0)$ with negligible Zeeman splitting [3] (see main text). Supplementary Figure 3 shows the evolutions of the fitting parameters as a function of the top-gate voltage V_{TG} for the different temperature. As mentioned in the main text, the temperature dependence of the inelastic scattering time is given by $\tau_\Phi \propto T^{-p}$ and therefore $B_{\mathrm{SO}} \propto T^p$ where p depends on the inelastic mechanism. We obtain a linear variation of B_Φ with temperature, which indicates that inelastic scattering is dominated by electron-electron interaction (p=1) [4]. B_{SO} is found to be independent of temperature below 10K which is the range where the dielectric constant of $SrTiO_3$ is rather

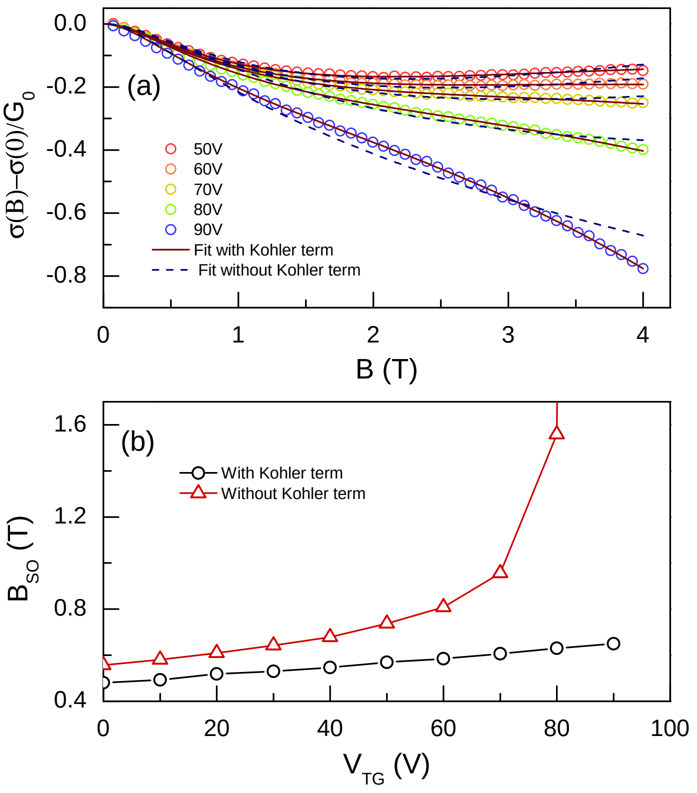

Figure S4: a) Magneto-conductance of the device for different positive values of V_{TG} fitted by the Maekawa-Fukuyama formula with and without the Kohler term. b) Evolution of the fitting parameters B_{SO} with and without the Kohler.

temperature independent. As the bending of the $SrTiO_3$ conduction band that defines the quantum well is not modified, the interfacial electric field E_z remains constant and the coupling constant α is not modified. The Kohler term is proportional to the square of the mobility which doesn't vary below 10K as it is also mainly determined by the confinement conditions. As a consequence, A_K doesn't change with temperature.

We emphasize here that the Kohler term is crucial in the analysis of the magnetoconductance data. As A_K increases quadratically with the mobility this term dominates the magneto-transport for positive gating. As shown in Supplementary Figure 4, fitting without this term leads to an incorrect determination of B_{SO}.

[1] N. Reyren et al. Science **317**, 1196–1199 (2007).

[2] P. D. Eerkes, W. G. van der Wiel. & H. Hilgenkamp, Appl. Phys. Lett. **103**, 201603 (2013).

[3] S. Maekawa, & H. J. Fukuyama, Phys. Soc. Jpn. **50**, 2516-2524 (1981).

[4] J. Biscaras *et al.*, Nature Communications **1**, 89 (2010).
